# Chinese Medicinal Herbs Targeting the Gut–Liver Axis and Adipose Tissue–Liver Axis for Non-Alcoholic Fatty Liver Disease Treatments: The Ancient Wisdom and Modern Science

**DOI:** 10.3389/fendo.2020.572729

**Published:** 2020-09-30

**Authors:** Shuwei Zhang, Yui-Tung Wong, Ka-Yu Tang, Hiu-Yee Kwan, Tao Su

**Affiliations:** ^1^ International Institute for Translational Chinese Medicine, Guangzhou University of Chinese Medicine, Guangzhou, China; ^2^ Centre for Cancer and Inflammation Research, School of Chinese Medicine, Hong Kong Baptist University, Hong Kong, China

**Keywords:** non-alcoholic fatty liver disease, pathogenesis, Chinese medicinal herbs, gut–liver axis, adipose tissue–liver axis

## Abstract

Non-alcoholic fatty liver disease (NAFLD) is one of the most common chronic liver diseases worldwide. The pathogenesis of NAFLD is complex. Frontline western medicines only ameliorate the symptoms of NAFLD. On the contrary, the uniqueness of Chinese medicine in its interpretation of NAFLD and the holistic therapeutic approach lead to a promising therapeutic efficacy. Recent studies reveal that the gut–liver axis and adipose tissue–liver axis play important roles in the development of NAFLD. Interestingly, with advanced technology, many herbal formulae are found to target the gut–liver axis and adipose tissue–liver axis and resolve the inflammation in NAFLD. This is the first review summarizes the current findings on the Chinese herbal formulae that target the two axes in NAFLD treatment. This review not only demonstrates how the ancient wisdom of Chinese medicine is being interpreted by modern pharmacological studies, but also provides valuable information for the further development of the herbal-based treatment for NAFLD.

## Introduction

**Figure 1:**
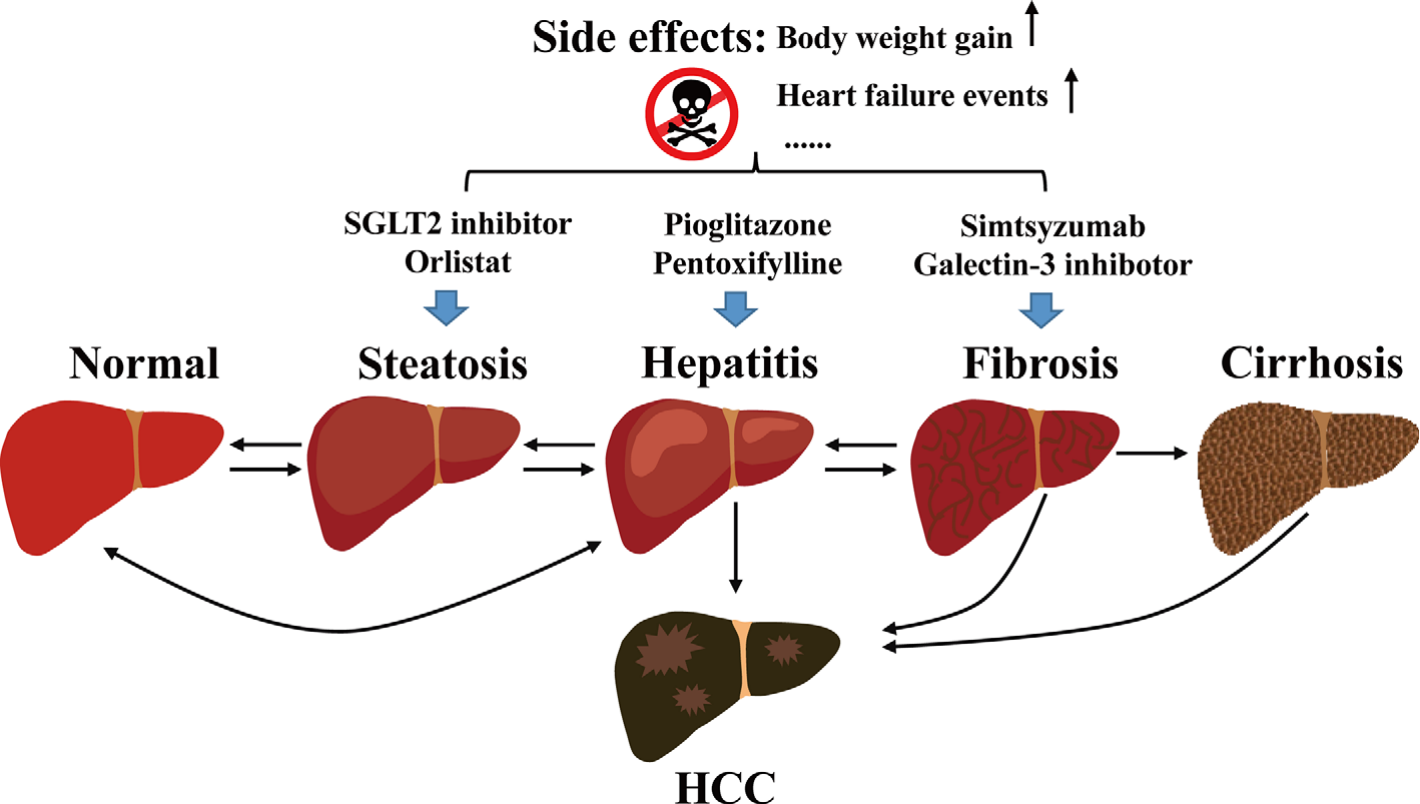
Development of NAFLD and the current management.

Non-alcoholic fatty liver disease (NAFLD) is a chronic liver disease. It develops from hepatic fatty infiltration known as non-alcoholic fatty liver (NAFL), to the stage of nonalcoholic steatohepatitis (NASH) with inflammatory cell infiltration accompanied with various degrees of fibrosis (1). NAFLD may eventually develop into hepatocellular carcinoma (HCC) (2, 3) (**Figure 1**).

Around 17–51% of adults in countries like China, France, Germany, Italy, Japan, Spain, UK, and US have developed NAFLD (4). The number of NAFLD cases with end-stage disease is expected to increase in these countries. Among them, China will have the highest incidence of NAFLD that may reach 314.58 million cases in 2030 (5); and the NASH population will also increase by 48% to 48.26 million cases or 15% of all the NAFLD cases (5). People with NAFLD have increased mortality. In particular, the liver-related mortality such as cirrhosis and HCC are markedly increased in the NASH population (6). Liver-related deaths in 2016 ranged from 2,490 in France to 30,240 in the US; it is predicted that China will surpass these countries and will have the largest number of liver-related deaths by 2030 (5). Hence, a better understanding of the pathogenesis of NAFLD is essential for the design of new and effective therapeutic interventions.

## The Pathogenesis of NAFLD Is Complex

The pathogenesis of NAFLD involves a complex interaction between obesity, environmental factors, and the gut microbiota (7). It is generally believed that changes in lifestyle and the global pandemic of obesity account for the rapid increase in the NAFLD prevalence in these countries (8). In particular, the western style dietary habit, such as high consumption of high-energy and processed foods (9). These foods are often referred as “junk food”, which are rich in monosaccharide, saturated fats and trans fats, and lack of nutrients (9, 10). Studies have demonstrated that the experimental animals exposed to “junk foods” could develop NAFLD within 10 weeks (11–13).

Many clinical and experimental studies have already revealed that NAFLD and NASH patients have elevated oxidative DNA damage in the liver (14, 15), which is due to the enhanced production of reactive oxygen species (ROS). ROS are the by-products of metabolism in the hepatocytes. Hepatic fatty acid accumulation promotes ROS generation because mitochondrial respiratory chain activity cannot couple with the enhanced mitochondrial fatty acid oxidation. Besides, liver specimens from NAFLD patients are also characterized by the elevated phosphorylation of endoplasmic reticulum (ER) stress marker such as eukaryotic initiation factor 2 (eIF2) (16), suggesting ER stress also contributes to the pathogenesis of NAFLD. These pathways can participate in the adaptive response of lipid accumulation and promote the production of ROS and oxidative stress (17). It is noteworthy that CYP2E1 and CYP4A have been considered as major sources of oxidative stress (18). Subsequently, oxidative stress leads to DNA damage, phospholipid membrane disruption by lipid peroxidation, and secretion of proinflammatory cytokines (19). Autophagy, which is an important degradative cellular pathway of the autodigestion of cellular proteins and organelles to obtain energy, has been suggested to have critical functions in both hepatocytes and nonparenchymal cells (i.e., macrophages and hepatic stellate cells) influencing insulin sensitivity, lipid accumulation, hepatocellular injury, and the innate immune response (20). In addition, insulin resistance can be acquired *via* multiple mechanisms, and may affect various steps in the insulin signaling cascade which finally suppresses important metabolic pathways causing overproduction of glucose, and ultimately result in liver cell damage and death, and hence accelerates NAFLD progression (21). Kupffer cells in the liver are capable to release large amounts of tumor necrosis factor (TNF) and interleukin-6 (IL-6), which directly induce hepatic inflammation and fibrogenesis, releasing several proinflammatory cytokines that are pathogenetic in NAFLD (22). Hence, understanding the pathogenesis of NAFLD is necessary for the identification of biomarkers for the prevention and treatment of the disease.

## The Gut–Liver Axis in NAFLD

Recent studies discover that alteration in the gut–liver axis is closely associated with the progression of NAFLD in the patients (23). Changes in the gut microbiome promote NAFLD development. In NAFLD patients, bacterial families Prevotellaceae and Enterobacteriaceae Proteobacteria, Enterobacteriaceae, and Escherichia are increased (24); while Oscillobacter, Bacteroidetes and Clostridium leptum (family Clostridiaceae) are reduced (25, 26). Patients with NASH have increased Ruminococcus, Blautia, and Dorea abundance, but a lower rate of Bacteroidetes compared to steatosis and healthy controls (27). These data imply an association between the presence of these bacterial families and NASH development. Indeed, the Bacteroides abundance is directly correlated with NASH severity (28).

The gut flora modifies bile acid metabolism (29). The gut microbes produce enzymes that convert primary bile acids into secondary bile acids in the intestines. NAFLD or NASH patients have moderate elevations of total bile acid, besides, the bile acid compositions in these patients are also changed (30). Disturbance of the gut microbiota decreases the synthesis of secondary bile acids, which in turn decreases activation of nuclear receptors such as farnesoid X receptor (FXR), pregnane X receptor, Takeda G-protein-coupled bile acid protein 5 and vitamin D receptor. Dysregulation of these receptor can lead to the development of NAFLD (31–33). For example, activation of FXR represses hepatic *de novo* lipogenesis, stimulates fatty acid β-oxidation, and hence reduces hepatic lipid accumulation (34–36); whereas reduced FRX activity promotes NAFLD development. The gut microbiota also regulates the immune balance in the gut *via* different pathways. For example, the abundance of *Faecalibacterium prausnitzii* is reduced in the patients. *F. prausnitzii* is an anti-inflammatory commensal, its reduced abundance increases interleukin-10 (IL-10) secretion and reduces IL-12 and interferon-γ expressions (37). Besides, the thinned intestinal mucus layer and increased gut permeability will increase the leakage of bacterial components that could activate Toll-like receptors (TLRs) or NOD-like receptors (38). TLRs recognize the microbial molecules “pathogen-associated molecular patterns” such as lipopolysaccharide (LPS) or the damage-associated molecular pattern. Activation of TLRs increases the production of tumor necrosis factor-α (TNF-α), IL-6, IL-8, and IL-12. In TLR-4 null mice, hepatic fat deposition is reduced, and NASH development is slowed down (39–41). The intestinal permeability is controlled by several multiprotein adhesive complexes including tight junctions, subjacent adherens junctions and desmosomes (42, 43). Studies have demonstrated that NAFLD is associated with increased gut permeability and the increased permeability appears to be caused by disruption of intercellular tight junctions (44). The increased gut permeability in these patients further promotes the inflammatory responses (45). Experimental study shows that in high-fat diet-fed mice, the gut becomes more permeable to the translocation of LPS; therefore, LPS can reach the liver through the portal vein and exacerbate liver inflammation and fibrosis (23, 46).

**Figure 2:**
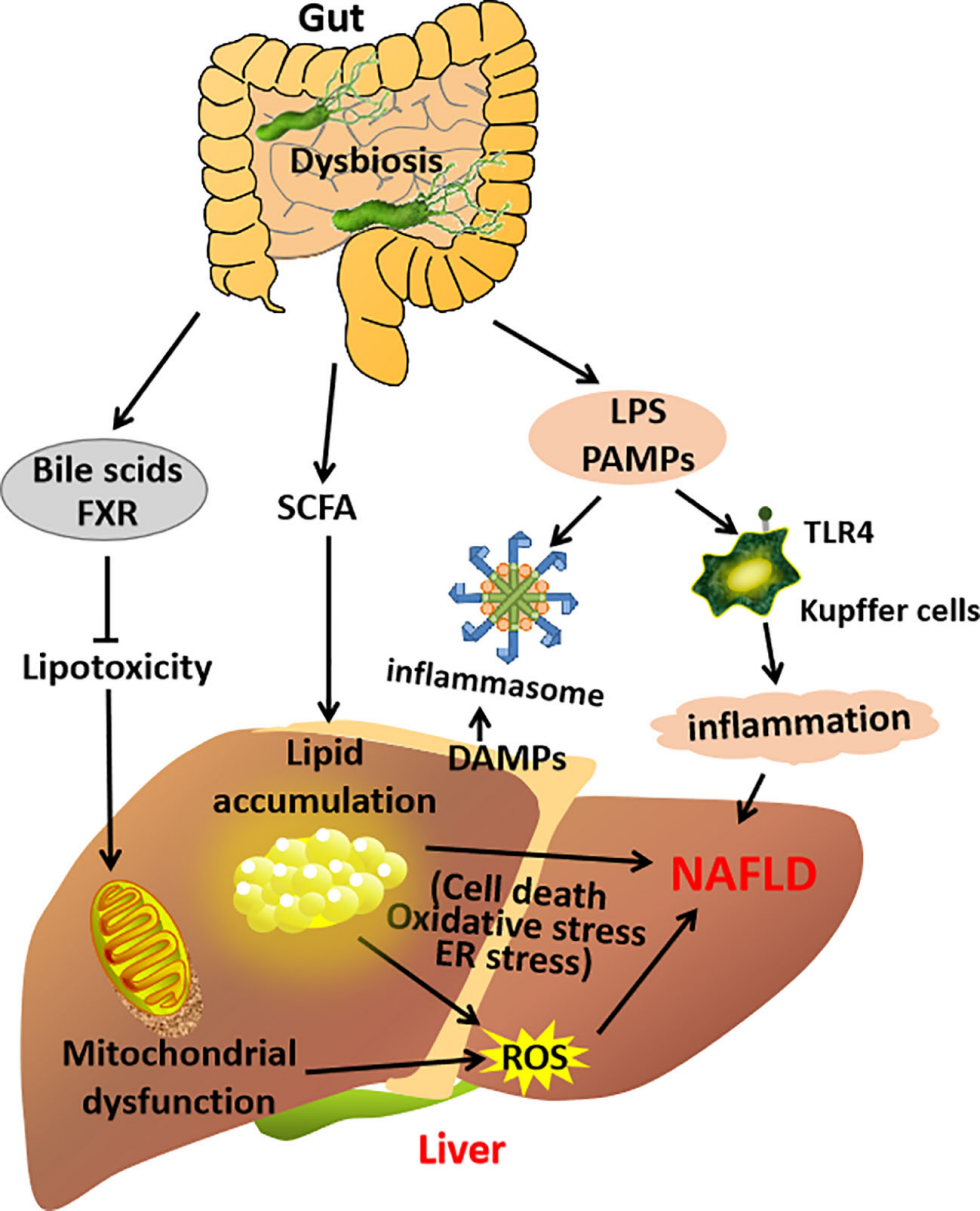
A schematic diagram demonstrating the contribution of the gut–liver axis in NAFLD pathogenesis.

All these clinical and experimental studies suggest that the gut–liver axis will trigger the proinflammatory and profibrogenetic pathways in the liver and promote NAFLD development (23, 46) (**Figure 2**).

## The Adipose Tissue–Liver Axis in NAFLD

The adipose tissue–liver axis also contributes to the NAFLD pathogenies. Adipose tissues in NAFLD patients have elevated expressions of inflammatory mediators (47). Besides, the adipose tissues may have enhanced infiltrate of macrophages. Clinical studies show that the severity of macrophage infiltration in the adipose tissue in the patients is directly correlated with the degree of hepatic steatosis, inflammation and fibrosis (48, 49). Besides, adipocyte hypertrophy leads to adipocyte cell death (50, 51), which alters the pro-inflammatory cytokine production and secretion (52). For example, the increased secretion of TNF-α (53) will induces hepatocyte death and modulates hepatic immune function. Clinical studies also suggest that TNF-α is a predictor of NASH and its level is directly related to the disease stages (54–57).

Adipose tissues are a source of lipids. However, dysfunctional adipose tissues will have limited fat storage capacity thus promoting the deposition of ectopic fat in liver and muscle (58), which is known as lipotoxicity. Lipotoxicity induces hepatic mitochondrial oxidative stress (59), which further accelerates the progression of NAFLD.

The dysfunctional adipose tissues will also alter adipokine production (60). Adiponectin and leptin are the two common adipokines. Adiponectin has anti-fibrotic effect in the liver (61), mediated by AMPK activity (62). Adiponectin also has anti-inflammatory effect, it blocks the activation of NF-κB, increases secretion of anti-inflammatory cytokines, and reduces the release of pro-inflammatory cytokines such as TNF-α and IL-6 (63). Therefore, decreased adiponectin level is associated with the advanced stage of the disease, it is considered as a predictor of the necro-inflammatory grade and fibrosis in NAFLD (64–66). Clinical studies have reported that reduced adiponectin and increased leptin levels result in hepatic steatosis and activation of inflammation and fibrogenesis (29). In mouse model, delivery of adiponectin improves steatohepatitis in the mouse models (67).

**Figure 3:**
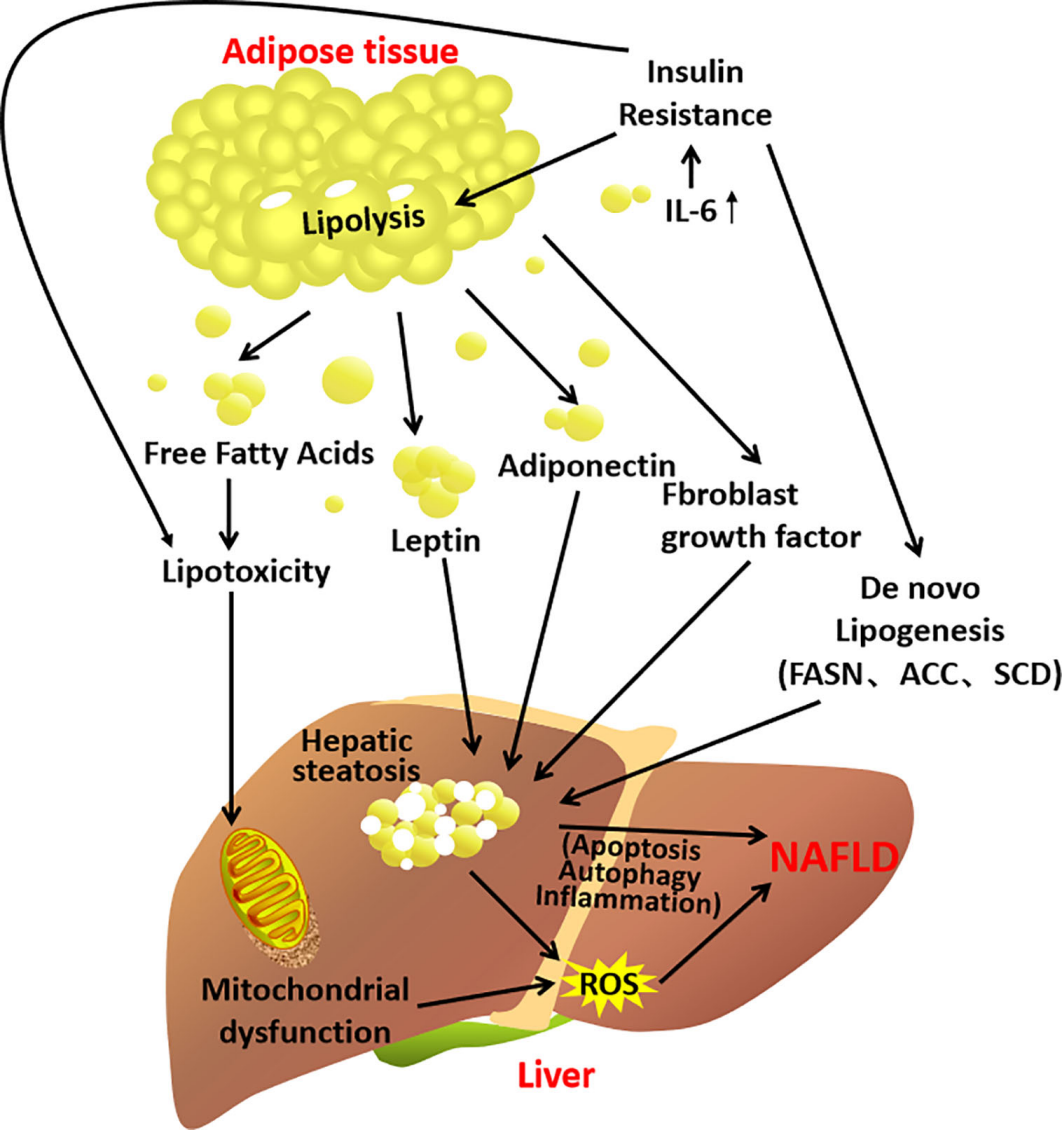
A schematic diagram demonstrating the contribution of the adipose tissue–liver axis in NAFLD pathogenesis.

Experimental study shows that removal of inflamed white adipose tissue indeed attenuates the development of NASH in the mice (68). Clinical studies have shown that the expression of adipocytokines in NAFLD patients is different from that in healthy people (56, 69, 70). Compared to the healthy, levels of leptin, TNF-α and IL-6 are significantly elevated in NAFLD patients, whereas the adiponectin level is significantly reduced. Therefore, adipocyte hypertrophy, adipocytes dysfunction and the subsequent inflammation in the adipose tissue–liver axis contribute to the development of NAFLD (**Figure 3**).

## Clinical Challenges in NAFLD Treatment

Current managements of NAFLD or NASH include diet regimen, aerobic exercise, and interventions that target the associated metabolic abnormalities (71, 72). For example, diabetic drug pioglitazone reduces NASH and the mean fibrosis score in patients with biopsy-proven NASH, regardless of the diabetes status (1). However, pioglitazone causes weight gain (73) and increases heart failure events (74), it is not an ideal medication for NAFLD patients. Furthermore, given the complicated pathogenesis, modifying lifestyle and dietary intervention may not always be effective to alleviate or ameliorate the NAFLD conditions. The current management of NAFLD is illustrated in **Figure 1**.

Studies also try to explore the beneficial effects of various dietary supplements. A clinical study shows that vitamin E, an anti-inflammatory medication, improves the NASH condition when compared with placebo in a randomized study of non-diabetic NAFLD individuals (75). Similar result is also demonstrated in a meta-analysis study which suggests vitamin E improves the histologic features of NASH, but not fibrosis (76). Besides, polyunsaturated fatty acids (PUFAs) have also been postulated to mitigate NAFLD and NASH. Experimental studies suggest that PUFAs activates hepatic peroxisome proliferator-activated receptor alpha and promotes fatty acid oxidation (77); downregulates sterol regulatory element binding protein-1 and reduces lipogenesis (78). PUFAs also reduces the production of pro-inflammatory mediators such as interleukin-6 (IL-6) and tumor necrosis factor (79). A meta-analysis study suggests that omega-3 PUFA supplementation significantly reduces hepatic lipid contents (80–82). However, the supplementation does not improve the NASH condition (82). All these studies suggest that taking dietary supplement is not an effective approach to reverse the NAFLD or NASH conditions.

## Combining Ancient Wisdom and Modern Science to Explore Herbal-Based Therapeutics for NAFLD Treatment

Nowadays, modern pharmacological and experimental studies have elucidated the mechanisms of action of Chinese herbal medicine in the treatment of NAFLD. In a meta-analysis study, twenty herbs that are commonly used for treating NAFLD have been identified (83). Among them, the top eight frequently used herbs and their constituting anti-NAFLD active compounds are summarized in **Table 1**.

**Table 1:** The top eight commonly used herbs and their constituting anti-NAFLD active compounds.

Herbs	Active compounds	Mechanisms underlying the anti-NAFLD effects	References
Crataegi Fructus (Shanzha)	Chlorogenic acid	Improve the adiponectin level; ameliorate liver injury and insulin resistance	(84–86)
	Hyperoside	Improve the adiponectin level	(84)
	Oleanolic acid	Stimulate AMPK phosphorylation and inhibit lipogenesis; improve insulin sensitivity	(87, 88)
Salviae Miltiorrhizae radix et Rhizoma (Danshen)	Salvianolic acid A	Decrease the hepatotoxic levels of cytokines and inhibit the excessive production of ROS	(89)
	Salvia miltiorrhiza polysaccharide	Decrease the expression levels of proinflammatory factors (eg. TNF-α, IL-6)	(90)
	Tanshinone IIA	Inhibit the TLR4/NF-κB signaling pathway	(91, 92)
	Salvianolic acid B	Increase the expression levels of tight junction protein occludin and ZO-1	(93)
Poria (Fuling)	Polysaccharide	Increase butyrate-producing bacteria Lachnospiracea and improve the gut mucosal integrity	(94)
	Poricoic acid	Ameliorate liver steatosis through the activation of AMPK phosphorylation	(95)
Alismatis rhizoma (Zexie)	Alisol B 23-acetate	Reduce hepatic triglyceride accumulation *via* FXR activation	(96, 97)
	Alisol A 24-acetate	Inhibit inflammatory cytokines (eg. TNFα, IL-6 levels); inhibit oxidative stress and induce autophagy	(98, 99)
Bupleuri Radix (Chaihu)	Saikosaponin A	Induce autophagy	(100, 101)
*Atractylodes macrocephala* Koidz. (Baizhu)	Atractylodes macrocephala polysaccharide	Activate the AMPK signaling pathway	(102)
	Atractylenolide III	Increase the phosphorylation of AMPK	(103)
Cassiae Semen (Juemingzi)	Obtusin	Increase the expression of intestinal tight-junction protein occludin and ZO-1; improve the intestinal mucosal barrier function	(104, 105)
	Emodin	Inhibit the expression levels of proinflammatory cytokines	(106, 107)
Curcumae Radix (Yujin)	Curcumin	Attenuate the hepatic steatosis via the Nrf2-FXR-LXR pathway; improve intestinal barrier function; decrease the expression levels of proinflammatory cytokines	(108, 109)

**Figure 4:**
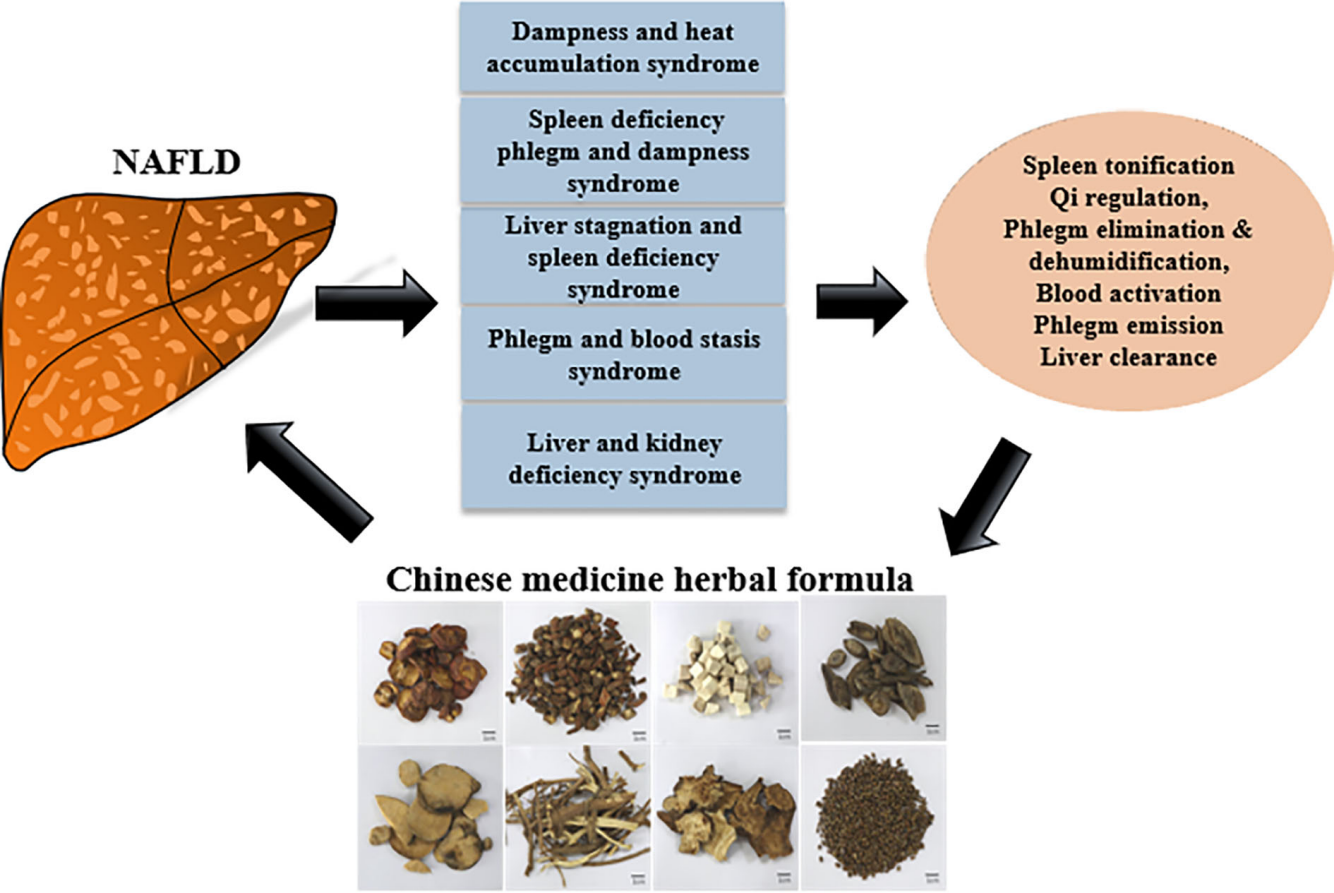
A schematic diagram demonstrating the treatment of NAFLD with Chinese medicine.

The diagnosis and interpretation for NAFLD in Chinese medicine is different from Western medicine. In Chinese medicine, NAFLD is further subdivided into syndromes (**Figure 4**), and a holistic therapeutic approach is employed to treat the patients. For example, the therapies include spleen tonification and qi regulation, phlegm elimination and dehumidification, blood activation and phlegm emission and liver clearance (110).

Many clinical studies show that Chinese herbal formulae are effective for NAFLD treatment as assessed by the clinical outcomes such as the NAFLD activity score (NAS) and hepatic or serum lipid levels. For example, a clinical trial has been done to study the effectiveness of Qu Yu Hua Tan Tong Luo (QYHTTL) in NAFLD treatment. QYHTTL comprises of Bupleuri Radix, Scutellariae Radix, Pinelliae Rhizoma, Codonopsis Radix, Glycyrrhizae Radix et Rhizoma, Jujubae Fructus, RJ, Morindae Officinalis Radix, and Oldenlandia Diffusa. The study demonstrates that QYHTTL significantly lowers the levels of hepatic aminotransferases and improves lipid profiles (111). Besides, a meta-analysis including 13 studies involving 1429 patients shows that another herbal formula, Huo Xue Hua Yu (HXHY) significantly reduces total cholesterol and triglyceride levels when compared to conventional treatment. HXHY also has a greater beneficial effect on liver function in reducing alanine transaminase (ALT) and aspartate transaminase (AST) (112). The main constituent herbs in HXHY are Salviae Miltiorrhizae radix et Rhizoma, Chuanxiong Rhizoma, Crataegi Fructus, Curcumae Longae Rhizoma, Curcumae Radix, Notoginseng Radix et Rhizoma, Persicae Semen, *Sparganii Rhizoma*, *Curcuma zedoaria Rhizoma*, Carthami Flos, Eupatorii Herba, Corydalis Rhizoma and Vaccariae Semen. However, the mechanisms of action underlying the therapeutic effects of these herbal formula are less studied.

### Experimental Studies Reveal That Many of the Herbal Formulae Regulate Hepatic Lipid Metabolism to Alleviate the NAFLD Condition

Since NAFLD has been described in 1980s, lots of experimental studies and clinical trials in China have been done to study the efficacies of TCMs for treating NAFLD. A Chinese herbal formula, Jiang Zhi Granule (JZG), is composed of Gynostemmatis Herba, Nelumbinis Folium, Salviae Miltiorrhizae radix et Rhizoma, Polygoni Cuspidati Rhizoma et Radix and Artemisiae Scopariae Herba (113). It is clinically safe, no adverse effect has been reported (114). In a clinical trial of 220 NAFLD patients, prescription of JZG significantly reduces body weight and improves hepatic steatosis (114). As the effectiveness of Chinese herbal formulas depends on the synergistic effects between multiple compounds and their targets, systems pharmacology approach has been used to explore the pharmacological mechanisms of JZG. Systems pharmacology is a novel strategy that can clarify the synergistic effects and the mechanisms of multi-component and multi-targeted agents (115). It can predict the active compounds within the herbal formula JZG and their corresponding therapeutic targets and synergistic effects. The analysis has highlighted a number of signaling pathways that may be involved in therapeutic effects of JZG, they are ER-phagosome pathway, proteasomal ubiquitin-dependent protein catabolic process, TRAF6-mediated induction of pro-inflammatory cytokines, MAPK cascade, regulation of lipid metabolism including steroid biosynthesis, cholesterol transport and fatty acid metabolism (116). Indeed, mechanistic study also shows that JZG regulates lipid metabolism. JZG significantly inhibits sterol regulatory element binding protein-1c (SREBP-1c) activity by inhibiting the liver X receptor-α (LXRα)-mediated SREBP-1c transcription and LXRα-independent SERBP-1c maturation (117, 118). It is well-known that LXRα trans-activates SREBP-1c (119), and SREBP-1c is involved in the pathogenesis of NAFL and NASH. Indeed, SREBP-1c is a critical transcription factor in lipogenesis, it initiates the transcription of lipogenic genes which enzymes cause lipid deposition. SREBP-1c also plays a role in ER stress. Study shows that AMP-activated protein kinase (AMPK)-mediated down-regulation of SREBP-1c alleviates the ER stress response in hepatocytes by suppressing the mechanistic target of rapamycin complex 1 signaling (120). Other experimental study demonstrates that JZG activates autophagy progression by inducing autophagosomes or co-localization of autophagosomes and lysosomes (121). Degradation of autolysosomes protects the hepatocytes against palmitic acid-induced injury. JZG also protects mitochondrial integrity against oxidative stress in the hepatocyte model (121). In the mouse model of NAFLD, JZG reduces hepatic lipid content and ameliorates inflammation, improves metabolic disorder and reduces liver injury (116, 118, 121). In CCl4-induced liver injury in NAFLD rat, JZG also significantly reduces hepatic inflammation, ER stress, hepatic necrosis, hepatic lipid, blood transaminases and blood lipids (122). Further delineation suggests that protopanaxadiol, tanshinone IIA, and emodin in the JZG formula significantly reduce hepatic lipid content while protopanaxadiol reduces the oxidative stress in the steatotic liver cell models (113). All these studies strongly suggest that JZG can be developed as herbal-based therapeutics as an adjunct treatment for NAFLD, by regulating the lipid metabolism, inducing autophagy and reducing hepatic inflammation.

Besides, experimental studies have revealed many other herbal formulae that can directly regulate hepatic lipid metabolism in the NAFLD subjects to alleviate the conditions. For example, Jiangzhi Capsule has been clinically used for the management of lipid abnormalities. It is composed of Astragali Radix, Poria, Nelumbinis Folium, Alismatis Rhizoma, Crataegi Fructus, Chaenomelis Fructus, Salviae Miltiorrhizae Radix et Rhizoma, Notoginseng Radix et Rhizoma, Typhae Pollen, Polygoni Cuspidati Rhizoma et Radix, Taraxaci Herba, Polygoni Multiflori Radix and Ligustri Lucidi Fructus. A recent study in NAFLD rat model shows that Jiangzhi Capsule downregulates fructose-stimulated hepatic overexpression of SREBP-1a and 1c, and hence the hepatic expressions of acetyl-CoA carboxylase-1, stearoyl-CoA desaturase-1 and acyl-coenzyme A: diacylglycerol acyltransferase (DGAT)-2, suggesting that Jiagzhi capsule possesses therapeutic effect on NALFD treatment by modulating the lipid metabolism (123). A classical traditional Chinese medicine formula known as Da Chai Hu Decoction (DCHD) is shown to reduce the levels of total cholesterol and triglyceride in the NAFLD rat models (124). Besides, DCHD also reduces the expressions of proinflammatory mediators such as transforming growth factor-β1 (TGF-β1), TLR4, TNF-α and nuclear factor-kappa B (NF-кB), suggesting DCHD is a potential therapeutics for the treatment of NAFLD by reducing hepatic lipids and resolving the hepatic inflammation (124). Another study shows that a Chinese herbal formula Chai Hu Li Zhong Tang (CHLZT) significantly reduces serum levels of total cholesterol and triglyceride in the NAFLD rat models, the treatment also significantly reduces the numbers of lipid droplets in the liver tissues and also in HepG2 liver cell models (125). Further mechanistic study suggests that CHLZT increases the levels of p-AMPKα and PPARγ in the NAFLD liver tissues and HepG2 cells, while decreasing the expressions and activities of ACC, SERBP-2 and 3-hydroxyl-3-methylglutaryl-coenzyme A reductase (125), suggesting that CHLZT improves the NAFLD condition by regulating the hepatic lipid metabolism.

### Herbal Formulae Modulate the Gut–Liver Axis in NAFLD Treatment

Other formulae can modulate the gut–liver axis for the NAFLD treatment. Si Ni San (SNS) is first recorded by Zhong-Jing Zhang during the Eastern Han Dynasty. In Chinese medicine theory, SNS coordinates the functions of liver and spleen, and has been used for thousands of years. SNS consists of Bupleuri Radix, Paeoniae Alba Radix, Aurantii Immaturus Fructus, and honey-fried Glycyrrhizae Radix et Rhizoma in equal proportions. In Chinese medicine, SNS is used to “dispel cold and cause restoration upon collapse”, it eliminates interior heat and relieves stagnation. Although SNS has not been used to treat NAFLD patient, it has been tested for its therapeutic effect on NAFLD in animal models. In NAFLD rat models, SNS treatment significantly reduces hepatic total cholesterol, triglyceride and free fatty acid levels (126). The treatment also has limited toxicity to the mice as it does not elevate the serum levels of aspartate aminotransferase and alanine aminotransferase (126). Similar results are observed in the NAFLD mouse models, SNS significantly reduces body weight, liver index, liver triglyceride level, visceral fat index, serum ALT and liver TNF-α levels (127). In the clinical cases, high systemic level of TNF-α is associated with the severity of NAFLD in morbidly obese patients (128). Indeed, TNF-α plays a pivotal role in the development and progression of NAFLD. In mouse model, TNF-α increases the expression of monocyte chemotactic protein-1 (Mcp1), TGF-β1, and tissue inhibitor of metalloproteinase-1 (Timp1) in hepatocytes (129). MCP1, TGFB1 and TIMP1 are all involved in the development of hepatic fibrogenesis. Knockout of TNF in mice improves glucose tolerance and significantly reduces the prevalence of hepatic steatosis (20% vs. 100%, *p*<0.0001) and fibrosis (15% vs. 65%, p=0.0057) (129). Furthermore, SNS may also control the development of NAFLD *via* the gut–liver axis. A study with NAFLD mouse model has been done to examine the changes in the gut microbiota after SNS treatment. The data show that gut bacterial composition and functions in the NAFLD mice are changed after SNS treatment. PICRUSt (Phylogenetic Investigation of Communities by Reconstruction of Unobserved States) reveals that the abundance of Oscillospira (genus level), Ruminococcaceae (family level), Clostridiales (order level), and Clostridia (class level) are higher in the SNS treatment group compared to control group (127). LEfSe (Linear discriminant analysis Effect Size) method is then used to determine the features (organisms, clades, operational taxonomic units, genes, or functions) that most likely to explain differences between classes by coupling standard tests for statistical significance with additional tests encoding biological consistency and effect relevance. The LEfSe analysis suggests that carbohydrate metabolism is changed after SNS treatment (127), which may due to the changes in the abundance of Clostridia (130). High carbohydrate consumption is known to contribute to NAFLD (131). Fructose and sugar are the major mediators of NAFLD (132). Manipulating the carbohydrate metabolism or lipogenesis may help to alleviate the NAFLD conditions. Indeed, SNS treatment significantly reduces the liver triglyceride contents in the NAFLD mouse models (127), which may due to reduced lipogenesis. However, further investigation is needed to validate the involvement of Clostridia in the SNS-mediated effects on NAFLD.

Besides, Shen Ling Bai Zhu powder (SLBZP) is also found to ameliorate NAFLD condition by manipulating the gut–liver axis. In NAFLD rat models, SLBZP reduces the serum level of total cholesterol and alleviates hepatic steatosis (133). Microbiome analysis reveals that SLBZP changes the intestinal microbiota. SLBZP-treated rats have increased abundance of short-chain fatty acid-producing bacteria such as Bifidobacterium and Anaerostipes when compared to control rats (133). During the development of NAFLD, SCFAs can bind to the G-protein-coupled receptor GPR43, which helps to maintain normal intestinal permeability and reduce mucosal inflammation. Interestingly, a clinical study suggests that Bifidobacterium spp abundance is inversely associated with NAFLD severity, and Bifidobacteria may have a protective role against the development of NAFLD and obesity (134).

Hu Gan Qing Zhi tablet (HGQZT) has been used to alleviate NAFLD condition in clinical practice. Experimental studies also confirm that the HGQZT has lipid-lowering and anti-inflammatory effects in NAFLD subjects (135, 136). Recently, an experimental study suggests that HGQZT ameliorates the NAFLD condition by manipulating the gut microbiota. 16s rRNA gene sequencing analysis shows that abundance of 39 genera is significantly different after HGQZT treatment in the NAFLD rat models. HGQZT reduces the abundance of the Firmicutes/Bacteroidetes ratio and increases those that have been reported to relieve the NAFLD condition. For example, Ruminococcaceae, Bacteroidales_S24-7_group, Bifidobacteria, Alistipes, and Anaeroplasma. Besides HGQZT also reduces the abundance of those that promote NAFLD progression, such as Enterobacteriaceae, Streptococcus, Holdemanella, Allobaculum, and Blautia. By changing the intestinal microbial abundance, the function of mucosal barrier could be improved, hence, the inflammatory responses in the NAFLD subjects can be alleviated (137).

Another Chinese herbal formula, Hong-qi Jiang-zhi Fang (HJF) is found to improve the hepatic steatosis and alleviate HFD-induced endotoxemia, inhibit NLRP3 inflammasome activation and reduce cytokine release such as IL-1β and IL-18 (138). Interestingly it also improves intestinal barrier integrity and gut microbiota structure in these rats. After HJF treatment, 15 families, including Helicobacteraceae, Verrucomicrobiaceae and Enterobacteriaceae are significantly decreased in the NAFLD rat models (138). These bacterial families have been shown to promote the development of NAFLD by increasing liver fat deposition and inflammation.

### Herbal Formula Modulates the Adipose Tissue–Liver Axis in NAFLD Treatment

Given the adipose tissue–liver axis contributes to the development of NAFLD, targeting this axis may help to improve the NAFLD condition and slow down its progression. However, Chinese herbal formula that target at the adipose tissue–liver axis in NAFLD are less studied.

Qing Gan Zi Shen Tang (QGZST) is a famous Chinese herbal formula. It is clinically used for the treatment of hypertension, obesity, hyperlipidemia and insulin resistance. Experimental study shows that QGZST significantly decreases adipocyte size of the high-fat diet-fed rat, it also remarkably reduces the serum levels of cholesterol and triglyceride (139). Furthermore, QGZST also dramatically attenuates the production of proinflammatory cytokines and adiponectin from the adipocytes by stimulating the activities of Sirtuin-1 and Forkhead box protein-O1, while reducing the expression of PPAR-γ, C/EBP-α, fatty acid binding protein-4, acetylated nuclear factor-κB-p65 and protein-tyrosine phosphatase-1B (139). These results strongly suggest that QGZST can be used to treat adipocyte hyperplasia and the inflammation. Study with NAFLD animal model will further suggest the potential of QGZST in ameliorating the NALFD condition by reducing adipocyte hyperplasia and resolving the associated inflammation. Sobokchukeo-Tang (ST) is a well-known formula that is used to treat primary dysmenorrhea caused by blood stasis syndrome in China and Korea. It comprises Foeniculi Fructus, Zingiberis Rhizoma, Carthami Flos, Myrrha, Angelicae Sinensis Radix, Cnidii Fructus, Cinnammomi Cortex, Paeoniae Rubra Raidx, Typhae Pollen and Trogopterori Faeces. An experimental study shows that ST inhibits 3T3-L1 pre-adipocyte differentiation by reducing the expressions of peroxisome proliferator-activated receptor-γ (PPAR-γ) and CCAAT-enhancer-binding proteins-α (C/EBP-α), it also reduces triglyceride and leptin levels in the adipocytes, suggesting ST possesses anti-adipogenesis effect. Besides, ST also significantly inhibits TNF-α and IL-6 production in the LPS-treated macrophages compared with LPS stimulation alone, suggesting ST has anti-inflammatory effect in the macrophages (140). Inhibiting adipogenesis and resolving inflammation in the infiltrate macrophage can help to slow down the progression of NAFLD. A modified Sijunzi decoction CHF03 comprises Scutellariae Barbatae Herba, Rehmanniae Radix, and Smilacis Glabrae Rhizoma shows protective effect against NAFLD by reducing oxidative stress that arisen from lipotoxicity. The accumulation of lipids induces oxidative stress and apoptosis (141), which are crucial to the pathogenesis of lipotoxic injury in the liver and accelerates NAFLD progression. In the palmitic acid-treated liver cell models that mimic the condition under lipotoxicity, CHF03 significantly alleviates oxidative stress as indicated by the reduced expressions of GSH, GSH-px, MDA, SOD, and CAT, it also reduces the abundance of NF-κB proteins in the cell models, indicating CHF03 reduces inflammation and oxidative stress in the livers. Besides, hepatic expressions of SERBP-1 and fatty acid synthase are also reduced after the treatment. These data suggest that CHF03 might have a beneficial role in the prevention of hepatic steatosis by attenuating lipotoxicity-associated oxidative stress and reducing lipogenesis (142). Similarly, another herbal formula, Liuwei Dihuang (LWDH) also reduces oxidative stress in the obese rat model. The study demonstrates that LWDH lowers serum levels of C-reactive protein, GSH and TNF-α, suggesting LWDH can reduces the oxidative stress in the rats (143). However, studies with NAFLD rat model can better suggest whether LWDH can be used to treat NALFD by reducing the lipotoxicity-associated oxidative stress. Other formula may regulate the release and production of adipokines and hence improve the NAFLD condition. Visfatin is an adipokine. Many clinical studies show that serum visfatin concentration in the NAFLD subjects is significantly higher than in controls (144, 145), suggesting a pathogenic role of visfatin in NAFLD development. A Chinese herbal formula Zhi Yi Xiao (ZYX) is found to reduces the expression of visfatin in the liver of NAFLD rat models (106). Further studies are needed to further suggest the therapeutic role of ZYX in treating NAFLD.

## Perspective

Chinese medicine employs a holistic approach as its therapeutic strategy, and the formulation usually comprises herbs that are classified into four catalogues, they are the “emperor drug” that acts on the main cause in pathophysiology, “minister drug” that acts on the main or the second causes in the pathophysiology, “assistant drug” that strengthens the effects of emperor drug and reduces any toxic effect of the emperor and minister drugs, “messenger drug” that boosts the action of the herbs in the formulation. Due to the complexity, Chinese medicine theory is usually difficult to be comprehended. Therefore, analysis of the underlying mechanisms of action of the herbal formulae becomes difficult. Furthermore, the synergies between the herbs and the herbal compounds in the decoction may further enhance the therapeutic effects, which will not be known without experimental studies.

Modern technologies have been developed to elucidate the molecular mechanism and to identify the effective components of the Chinese herbal formulae. Computational systems biology such as connectivity map or multilayer map that consists of phenotype network, biological network and herbal network have been applied to study the therapeutic mechanisms of the herbal formulas (146–148). Metabolomics-based phenomics and the network construction can highlight the metabolic pathways that lead to the therapeutic effects of the herbal treatment. Ligand-based pharmacophore and molecular docking models can further suggest the therapeutic targets of the herbal formula. In addition, with the development of bioinformatics, the combination of traditional Chinese medicine and intestinal microbiology is of great significance for maintaining the health status of host–microorganism. Beside the gut microbiota, by comparing the differences of the microflora of the tongue coating between gastritis and healthy people, the tongue coating microbiome is also considered as potential biomarkers for diseases like gastritis including precancerous cascade (149).

**Figure 5:**
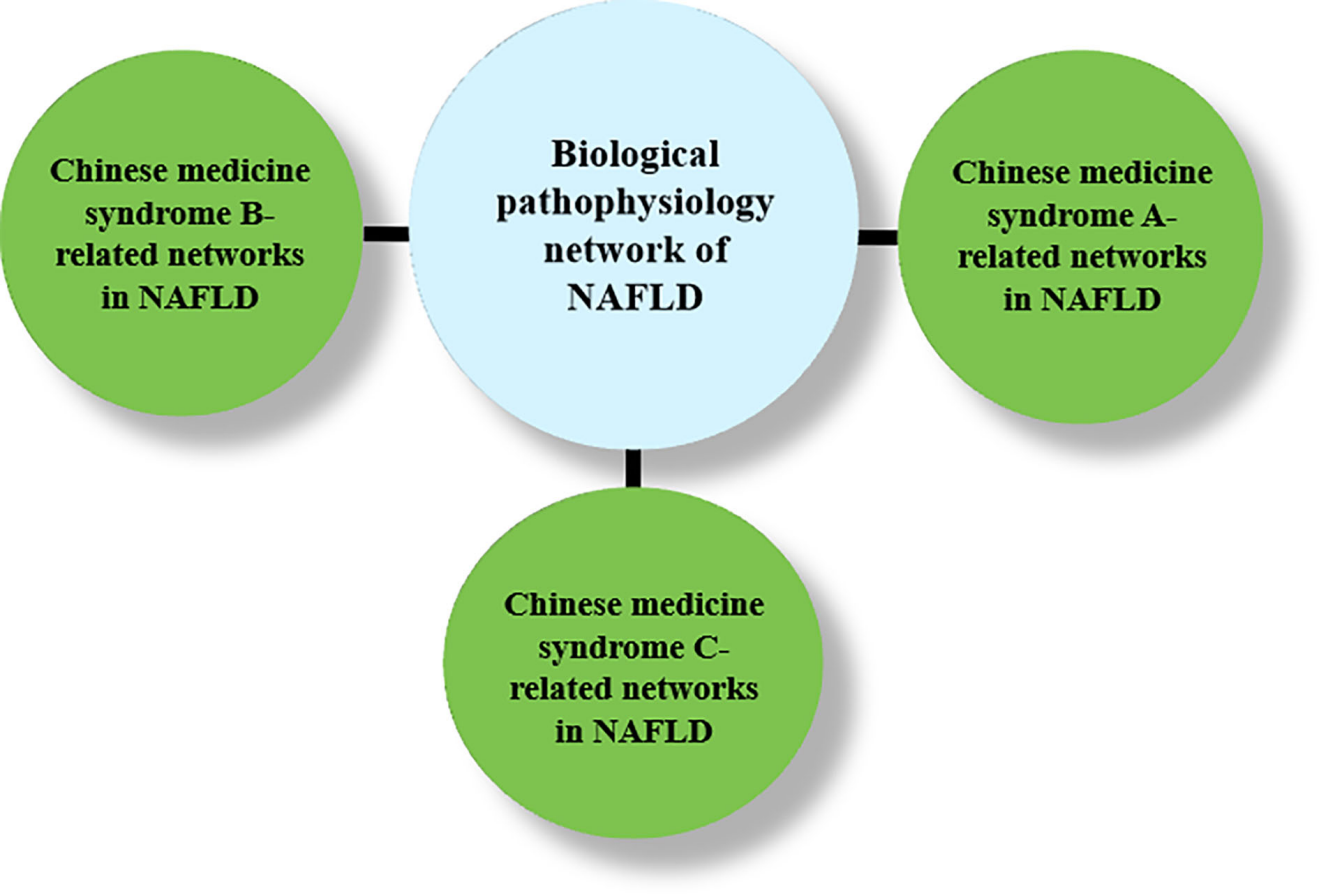
A schematic diagram of drug discovery for NAFLD treatment based on TCM-based network pharmacology.

The state-of-the-art technologies can also bring about the discovery of novel herbal-based therapeutics for the treatment. For example, computer-aid drug design (CADD) can be used to design novel herbal formula or to modify the existing formula (150). The Chinese medicine-based network pharmacology that comprises the Chinese medicine-syndrome network and the biological network of the disease is a new strategy of multiple-compound drug discoveries (147) (**Figure 5**). The combination of the Chinese medicine syndrome and the western pathophysiological understanding of NAFLD also demonstrates the integration of the ancient wisdom and the modern technology for the discovery of new drugs.

## Conclusion

The NAFLD pandemic implies an increase number of patients diagnosed with NAFL, NASH, liver fibrosis and HCC in the near future. Although the interpretation and diagnosis of NAFLD in Chinese medicine are different from those in western medicine, many clinical and experimental studies suggest that Chinese herbal formula are effective in reducing hepatic steatosis, and revolving inflammation *via* many mechanistic pathways. Some Chines herbal formulae also target at the gut–liver axis in NAFLD, while the adipose tissue–liver axis emerges to be a therapeutic approach for the treatment of NAFLD. More clinical and in-depth mechanistic investigations may help to validate the roles of these Chinese herbal formulae for the treatment of NAFLD. Application of the modern technology can also help to discover novel herbal-based therapeutics for the treatment.

## Author Contributions

H-YK and TS wrote and edited the manuscript. SZ, Y-TW, and K-YT participated in writing process. TS revised and finalized the manuscript. All authors contributed to the article and approved the submitted version.

## Funding

This work was partially supported by Research Grant Council of HKSAR HKBU-22103017-ECS, Innovation & Technology Commission #PRP/015/19FX, National Natural Science Foundation of China #SCM-2016-NSFC-003 and Natural Science Foundation of Guangdong Province #2018A0303130122 to H-YK; the National Natural Science Foundation of China 81703705, the Opening Project of Zhejiang Provincial Preponderant and Characteristic Subject of Key University (Traditional Chinese Pharmacology), Zhejiang Chinese Medical University (ZYAOX2018010) to TS.

## Conflict of Interest

The authors declare that the research was conducted in the absence of any commercial or financial relationships that could be construed as a potential conflict of interest.

## References

[1] Chalasani N Younossi Z Lavine JE Charlton M Cusi K Rinella M The diagnosis and management of nonalcoholic fatty liver disease: practice guidance from the American Association for the Study of Liver Diseases. Hepatology (2018) 67:328–57. 10.1002/hep.29367 28714183

[2] Vilar-Gomez E Calzadilla-Bertot L Wong VW-S Castellanos M Aller-de la Fuente R Metwally M Fibrosis severity as a determinant of cause-specific mortality in patients with advanced nonalcoholic fatty liver disease: a multi-national cohort study. Gastroenterology (2018) 155:443–57. 10.1053/j.gastro.2018.04.034 29733831

[3] Younes R Bugianesi E Should we undertake surveillance for HCC in patients with NAFLD? J Hepatol (2018) 68:326–34. 10.1016/j.jhep.2017.10.006 29122695

[4] Rinella ME Nonalcoholic fatty liver disease: a systematic review. Jama (2015) 313:2263–73. 10.1001/jama.2015.5370 26057287

[5] Estes C Anstee QM Arias-Loste MT Bantel H Bellentani S Caballeria J Modeling nafld disease burden in china, france, germany, italy, japan, spain, united kingdom, and united states for the period 2016–2030. J Hepatol (2018) 69:896–904. 10.1016/j.jhep.2018.05.036 29886156

[6] Musso G Gambino R Cassader M Pagano G Meta-analysis: natural history of non-alcoholic fatty liver disease (NAFLD) and diagnostic accuracy of non-invasive tests for liver disease severity. Ann Med (2011) 43:617–49. 10.3109/07853890.2010.518623 21039302

[7] Arab JP Arrese M Trauner M Recent insights into the pathogenesis of nonalcoholic fatty liver disease. Annu Rev Pathol: Mech Dis (2018) 13:321–50. 10.1146/annurev-pathol-020117-043617 29414249

[8] Younossi Z Anstee QM Marietti M Hardy T Henry L Eslam M Global burden of NAFLD and NASH: trends, predictions, risk factors and prevention. Nat Rev Gastroenterol Hepatol (2018) 15:11–20. 10.1038/nrgastro.2017.109 28930295

[9] Elmaogullari S Demirel F Hatipoglu N Risk factors that affect metabolic health status in obese children. J Pediatr Endocrinol Metab (2017) 30:49–55. 10.1515/jpem-2016-0128 27992361

[10] Sampey BP Freemerman AJ Zhang J Kuan PF Galanko JA O’Connell TM Metabolomic profiling reveals mitochondrial-derived lipid biomarkers that drive obesity-associated inflammation. PloS One (2012) 7:e38812. 10.1371/journal.pone.0038812 22701716 PMC3373493

[11] Gasparin FRS Carreño FO Mewes JM Gilglioni EH Pagadigorria CLS Natali MRM Sex differences in the development of hepatic steatosis in cafeteria diet-induced obesity in young mice. Biochim Biophys Acta (BBA) Mol Basis Dis (2018) 1864:2495–509. 10.1016/j.bbadis.2018.04.004 29653185

[12] Sampey BP Vanhoose AM Winfield HM Freemerman AJ Muehlbauer MJ Fueger PT Cafeteria diet is a robust model of human metabolic syndrome with liver and adipose inflammation: comparison to high-fat diet. Obesity (2011) 19:1109–17. 10.1038/oby.2011.18 PMC3130193 21331068

[13] Parafati M Lascala A Morittu VM Trimboli F Rizzuto A Brunelli E Bergamot polyphenol fraction prevents nonalcoholic fatty liver disease via stimulation of lipophagy in cafeteria diet-induced rat model of metabolic syndrome. J Nutr Biochem (2015) 26:938–48. 10.1016/j.jnutbio.2015.03.008 26025327

[14] Seki S Kitada T Yamada T Sakaguchi H Nakatani K Wakasa K In situ detection of lipid peroxidation and oxidative DNA damage in non-alcoholic fatty liver diseases. J Hepatol (2002) 37:56–62. 10.1016/S0168-8278(02)00073-9 12076862

[15] Tanaka S Miyanishi K Kobune M Kawano Y Hoki T Kubo T Increased hepatic oxidative DNA damage in patients with nonalcoholic steatohepatitis who develop hepatocellular carcinoma. J Gastroenterol (2013) 48:1249–58. 10.1007/s00535-012-0739-0 23329365

[16] Puri P Mirshahi F Cheung O Natarajan R Maher JW Kellum JM Activation and dysregulation of the unfolded protein response in nonalcoholic fatty liver disease. Gastroenterology (2008) 134:568–76. 10.1053/j.gastro.2007.10.039 18082745

[17] Robertson G Leclercq I Farrell GC II Cytochrome P-450 enzymes and oxidative stress. Am J Physiol-Gastrointest Liver Physiol (2001) 281:G1135–9. 10.1152/ajpgi.2001.281.5.G1135 11668021

[18] Bellanti F Villani R Facciorusso A Vendemiale G Serviddio G Lipid oxidation products in the pathogenesis of non-alcoholic steatohepatitis. Free Radical Biol Med (2017) 111:173–85. 10.1016/j.freeradbiomed.2017.01.023 28109892

[19] Sanyal AJ Campbell–Sargent C Mirshahi F Rizzo WB Contos MJ Sterling RK Nonalcoholic steatohepatitis: association of insulin resistance and mitochondrial abnormalities. Gastroenterology (2001) 120:1183–92. 10.1053/gast.2001.23256 11266382

[20] Czaja MJ Function of autophagy in nonalcoholic fatty liver disease. Digest Dis Sci (2016) 61:1304–13. 10.1007/s10620-015-4025-x PMC4838507 26725058

[21] Santoleri D Titchenell PM Resolving the paradox of hepatic insulin resistance. Cell Mol Gastroenterol Hepatol (2019) 7:447–56. 10.1016/j.jcmgh.2018.10.016 PMC6369222 30739869

[22] Hirosumi J Tuncman G Chang L Görgün CZ Uysal KT Maeda K A central role for JNK in obesity and insulin resistance. Nature (2002) 420:333–6. 10.1038/nature01137 12447443

[23] Pierantonelli I Svegliati-Baroni G Nonalcoholic fatty liver disease: basic pathogenetic mechanisms in the progression from NAFLD to NASH. Transplantation (2019) 103:e1–e13. 10.1097/TP.0000000000002480 30300287

[24] Zhu L Baker SS Gill C Liu W Alkhouri R Baker RD Characterization of gut microbiomes in nonalcoholic steatohepatitis (NASH) patients: a connection between endogenous alcohol and NASH. Hepatology (2013) 57:601–9. 10.1002/hep.26093 23055155

[25] Mouzaki M Wang AY Bandsma R Comelli EM Arendt BM Zhang L Bile acids and dysbiosis in non-alcoholic fatty liver disease. PloS One (2016) 11:e0151829. 10.1371/journal.pone.0151829 27203081 PMC4874546

[26] Del Chierico F Nobili V Vernocchi P Russo A De Stefanis C Gnani D Gut microbiota profiling of pediatric nonalcoholic fatty liver disease and obese patients unveiled by an integrated meta-omics-based approach. Hepatology (2017) 65:451–64. 10.1002/hep.28572 27028797

[27] Mouzaki M Comelli EM Arendt BM Bonengel J Fung SK Fischer SE Intestinal microbiota in patients with nonalcoholic fatty liver disease. Hepatology (2013) 58:120–7. 10.1002/hep.26319 23401313

[28] Grabherr F Grander C Effenberger M Adolph TE Tilg H Gut dysfunction and non-alcoholic fatty liver disease. Front Endocrinol (2019) 10:661–9. 10.3389/fendo.2019.00611 PMC6742694 31555219

[29] Tremaroli V Bäckhed F Functional interactions between the gut microbiota and host metabolism. Nature (2012) 489:242–9. 10.1038/nature11552 22972297

[30] Bechmann LP Kocabayoglu P Sowa JP Sydor S Best J Schlattjan M Free fatty acids repress small heterodimer partner (SHP) activation and adiponectin counteracts bile acid-induced liver injury in superobese patients with nonalcoholic steatohepatitis. Hepatology (2013) 57:1394–406. 10.1002/hep.26225 23299969

[31] Chen J Thomsen M Vitetta L Interaction of gut microbiota with dysregulation of bile acids in the pathogenesis of nonalcoholic fatty liver disease and potential therapeutic implications of probiotics. J Cell Biochem (2019) 120:2713–20. 10.1002/jcb.27635 30443932

[32] Parséus A Sommer N Sommer F Caesar R Molinaro A Ståhlman M Microbiota-induced obesity requires farnesoid X receptor. Gut (2017) 66:429–37. 10.1136/gutjnl-2015-310283 PMC5534765 26740296

[33] Liu L Liu Z Li H Cao Z Li W Song Z Naturally Occurring TPE-CA Maintains Gut Microbiota and Bile Acids Homeostasis via FXR Signaling Modulation of the Liver–Gut Axis. Front Pharmacol (2020) 11:12–27. 10.3389/fphar.2020.00012 32116693 PMC7015895

[34] Pineda Torra IS Claudel T Duval C Kosykh V Fruchart JC Staels B Bile acids induce the expression of the human peroxisome proliferator-activated receptor α gene via activation of the farnesoid X receptor. Mol Endocrinol (2003) 17:259–72. 10.1210/me.2002-0120 12554753

[35] Watanabe M Houten SM Wang L Moschetta A Mangelsdorf DJ Heyman RA Bile acids lower triglyceride levels via a pathway involving FXR, SHP, and SREBP-1c. J Clin Invest (2004) 113:1408–18. 10.1172/JCI21025 PMC406532 15146238

[36] Savkur RS Bramlett KS Michael LF Burris TP Regulation of pyruvate dehydrogenase kinase expression by the farnesoid X receptor. Biochem Biophys Res Commun (2005) 329:391–6. 10.1016/j.bbrc.2005.01.141 15721319

[37] Fukui H Role of gut dysbiosis in liver diseases: what have we learned so far? Diseases (2019) 7:58–99. 10.3390/diseases7040058 PMC6956030 31726747

[38] Miura K Ohnishi H Role of gut microbiota and Toll-like receptors in nonalcoholic fatty liver disease. World J Gastroenterol: WJG (2014) 20:7381–91. 10.3748/wjg.v20.i23.7381 PMC4064083 24966608

[39] Poggi M Bastelica D Gual P Iglesias M Gremeaux T Knauf C C3H/HeJ mice carrying a toll-like receptor 4 mutation are protected against the development of insulin resistance in white adipose tissue in response to a high-fat diet. Diabetologia (2007) 50:1267–76. 10.1007/s00125-007-0654-8 17426960

[40] Saberi M Woods NB de Luca C Schenk S Lu JC Bandyopadhyay G Hematopoietic cell-specific deletion of toll-like receptor 4 ameliorates hepatic and adipose tissue insulin resistance in high-fat-fed mice. Cell Metab (2009) 10:419–29. 10.1016/j.cmet.2009.09.006 PMC2790319 19883619

[41] Henao-Mejia J Elinav E Jin C Hao L Mehal WZ Strowig T Inflammasome-mediated dysbiosis regulates progression of NAFLD and obesity. Nature (2012) 482:179–85. 10.1038/nature10809 PMC3276682 22297845

[42] Krawczyk M Maciejewska D Ryterska K Czerwińka-Rogowska M Jamioł-Milc D Skonieczna-Żydecka K Gut permeability might be improved by dietary fiber in individuals with nonalcoholic fatty liver disease (NAFLD) undergoing weight reduction. Nutrients (2018) 10:1793–802. 10.3390/nu10111793 PMC6266494 30453660

[43] Lechuga S Ivanov AI Disruption of the epithelial barrier during intestinal inflammation: Quest for new molecules and mechanisms. Biochim Biophys Acta (BBA) Mol Cell Res (2017) 1864:1183–94. 10.1016/j.bbamcr.2017.03.007 PMC5507344 28322932

[44] Miele L Valenza V La Torre G Montalto M Cammarota G Ricci R Increased intestinal permeability and tight junction alterations in nonalcoholic fatty liver disease. Hepatology (2009) 49:1877–87. 10.1002/hep.22848 19291785

[45] Luther J Garber JJ Khalili H Dave M Bale SS Jindal R Hepatic injury in nonalcoholic steatohepatitis contributes to altered intestinal permeability. Cell Mol Gastroenterol Hepatol (2015) 1:222–32. 10.1016/j.jcmgh.2015.01.001 PMC4578658 26405687

[46] Gäbele E Dostert K Hofmann C Wiest R Schölmerich J Hellerbrand C DSS induced colitis increases portal LPS levels and enhances hepatic inflammation and fibrogenesis in experimental NASH. J Hepatol (2011) 55:1391–9. 10.1016/j.jhep.2011.02.035 21703208

[47] Du Plessis J Van Pelt J Korf H Mathieu C Van der Schueren B Lannoo M Association of adipose tissue inflammation with histologic severity of nonalcoholic fatty liver disease. Gastroenterology (2015) 149:635–48. 10.1053/j.gastro.2015.05.044 26028579

[48] Kolak M Westerbacka J Velagapudi VR Wågsäter D Yetukuri L Makkonen J Adipose tissue inflammation and increased ceramide content characterize subjects with high liver fat content independent of obesity. Diabetes (2007) 56:1960–8. 10.2337/db07-0111 17620421

[49] Tordjman J Poitou C Hugol D Bouillot JL Basdevant A Bedossa P Association between omental adipose tissue macrophages and liver histopathology in morbid obesity: influence of glycemic status. J Hepatol (2009) 51:354–62. 10.1016/j.jhep.2009.02.031 19464069

[50] Giordano A Murano I Mondini E Perugini J Smorlesi A Severi I Obese adipocytes show ultrastructural features of stressed cells and die of pyroptosis. J Lipid Res (2013) 54:2423–36. 10.1194/jlr.M038638 PMC3735940 23836106

[51] Hosogai N Fukuhara A Oshima K Miyata Y Tanaka S Segawa K Adipose tissue hypoxia in obesity and its impact on adipocytokine dysregulation. Diabetes (2007) 56:901–11. 10.2337/db06-0911 17395738

[52] Skurk T Alberti-Huber C Herder C Hauner H Relationship between adipocyte size and adipokine expression and secretion. J Clin Endocrinol Metab (2007) 92:1023–33. 10.1210/jc.2006-1055 17164304

[53] Lumeng CN Bodzin JL Saltiel AR Obesity induces a phenotypic switch in adipose tissue macrophage polarization. J Clin Invest (2007) 117:175–84. 10.1172/JCI29881 PMC1716210 17200717

[54] Diehl AM Tumor necrosis factor and its potential role in insulin resistance and nonalcoholic fatty liver disease. Clinics liver Dis (2004) 8:619–38. 10.1016/j.cld.2004.04.012 15331067

[55] Dongsheng C Minsheng Y Frantz Daniel F Melendez Peter A Lone H Jongsoon L Local and systemic insulin resistance resulting from hepatic activation of IKK-beta and NF-kappaB. Nat Med (2005) 11:183–90. 10.1038/nm1166 PMC1440292 15685173

[56] Jarrar M Baranova A Collantes R Ranard B Stepanova M Bennett C Adipokines and cytokines in non-alcoholic fatty liver disease. Aliment Pharmacol Ther (2008) 27:412–21. 10.1111/j.1365-2036.2007.03586.x 18081738

[57] Abenavoli L Peta V Role of adipokines and cytokines in non-alcoholic fatty liver disease. Rev Recent Clin Trials (2014) 9:134–40. 10.2174/1574887109666141216102458 25514909

[58] Szendroedi J Roden M Ectopic lipids and organ function. Curr Opin Lipidol (2009) 20:50–6. 10.1097/MOL.0b013e328321b3a8 19133412

[59] Galmés-Pascual BM Martínez-Cignoni MR Morán-Costoya A Bauza-Thorbrügge M Sbert-Roig M Valle A 17β-estradiol ameliorates lipotoxicity-induced hepatic mitochondrial oxidative stress and insulin resistance. Free Radical Biol Med (2020) 150:148–60. 10.1016/j.freeradbiomed.2020.02.016 32105829

[60] Abenavoli L Luigiano C Guzzi P Milic N Morace C Stelitano L Serum adipokine levels in overweight patients and their relationship with non-alcoholic fatty liver disease. Panminerva Med (2014) 56:189–93. 24994581

[61] Kamada Y Tamura S Kiso S Matsumoto H Saji Y Yoshida Y Enhanced carbon tetrachloride-induced liver fibrosis in mice lacking adiponectin. Gastroenterology (2003) 125:1796–807. 10.1053/j.gastro.y2003.08.029 14724832

[62] Adachi M Brenner DA High molecular weight adiponectin inhibits proliferation of hepatic stellate cells via activation of adenosine monophosphate–activated protein kinase. Hepatology (2008) 47:677–85. 10.1002/hep.21991 18220291

[63] Tilg H Moschen AR Adipocytokines: mediators linking adipose tissue, inflammation and immunity. Nat Rev Immunol (2006) 6:772–83. 10.1038/nri1937 16998510

[64] Musso G Gambino R Biroli G Carello M Fagà E Pacini G Hypoadiponectinemia predicts the severity of hepatic fibrosis and pancreatic Beta-cell dysfunction in nondiabetic nonobese patients with nonalcoholic steatohepatitis. Am J Gastroenterol (2005) 100:2438–46. 10.1111/j.1572-0241.2005.00297.x 16279898

[65] Handy JA Saxena NK Fu P Lin S Mells JE Gupta NA Adiponectin activation of AMPK disrupts leptin-mediated hepatic fibrosis via suppressors of cytokine signaling (SOCS-3). J Cell Biochem (2010) 110:1195–207. 10.1002/jcb.22634 PMC2907429 20564215

[66] Polyzos SA Aronis KN Kountouras J Raptis DD Vasiloglou MF Mantzoros CS Circulating leptin in non-alcoholic fatty liver disease: a systematic review and meta-analysis. Springer (2016) 59:30–43. 10.1007/s00125-015-3769-3 26407715

[67] Xu A Wang Y Keshaw H Xu LY Lam KS Cooper GJ The fat-derived hormone adiponectin alleviates alcoholic and nonalcoholic fatty liver diseases in mice. J Clin Invest (2003) 112:91–100. 10.1172/JCI200317797 12840063 PMC162288

[68] Mulder P Morrison M Wielinga P Van Duyvenvoorde W Kooistra T Kleemann R Surgical removal of inflamed epididymal white adipose tissue attenuates the development of non-alcoholic steatohepatitis in obesity. Int J Obes (2016) 40:675–84. 10.1038/ijo.2015.226 PMC4827008 26499443

[69] Kaser S Moschen A Cayon A Kaser A Crespo J Pons-Romero F Adiponectin and its receptors in non-alcoholic steatohepatitis. Gut (2005) 54:117–21. 10.1136/gut.2003.037010 PMC1774357 15591515

[70] Javor ED Ghany MG Cochran EK Oral EA DePaoli AM Premkumar A Leptin reverses nonalcoholic steatohepatitis in patients with severe lipodystrophy. Hepatology (2005) 41:753–60. 10.1002/hep.20672 15791619

[71] Francque S Vonghia L Pharmacological treatment for non-alcoholic fatty liver disease. Adv Ther (2019) 36:1052–74. 10.1007/s12325-019-00898-6 PMC6824365 30888594

[72] Alkhouri N Scott A An update on the pharmacological treatment of nonalcoholic fatty liver disease: beyond lifestyle modifications. Clin liver Dis (2018) 11:82–6. 10.1002/cld.708 PMC6370316 30761212

[73] Aghamohammadzadeh N Niafar M Dalir Abdolahinia E Najafipour F Mohamadzadeh Gharebaghi S Adabi K The effect of pioglitazone on weight, lipid profile and liver enzymes in type 2 diabetic patients. Ther Adv Endocrinol Metab (2015) 6:56–60. 10.1177/2042018815574229 25941563 PMC4406881

[74] Erdmann E Charbonnel B Wilcox RG Skene AM Massi-Benedetti M Yates J Pioglitazone use and heart failure in patients with type 2 diabetes and preexisting cardiovascular disease: data from the PROactive study (PROactive 08). Diabetes Care (2007) 30:2773–8. 10.2337/dc07-0717 17666462

[75] Sanyal AJ Chalasani N Kowdley KV McCullough A Diehl AM Bass NM or placebo for nonalcoholic steatohepatitis. New Engl J Med (2010) 362:1675–85. 10.1056/NEJMoa0907929 PMC2928471 20427778

[76] Said A Akhter A Meta-analysis of randomized controlled trials of pharmacologic agents in non-alcoholic steatohepatitis. Ann Hepatol (2017) 16:538–47. 10.5604/01.3001.0010.0284 28611274

[77] Arendt BM Comelli EM Ma DW Lou W Teterina A Kim T Altered hepatic gene expression in nonalcoholic fatty liver disease is associated with lower hepatic n-3 and n-6 polyunsaturated fatty acids. Hepatology (2015) 61:1565–78. 10.1002/hep.27695 25581263

[78] Takeuchi Y Yahagi N Izumida Y Nishi M Kubota M Teraoka Y Polyunsaturated fatty acids selectively suppress sterol regulatory element-binding protein-1 through proteolytic processing and autoloop regulatory circuit. J Biol Chem (2010) 285:11681–91. 10.1074/jbc.M109.096107 PMC2857043 20145241

[79] Zelber-Sagi S Salomone F Mlynarsky L The Mediterranean dietary pattern as the diet of choice for non-alcoholic fatty liver disease: Evidence and plausible mechanisms. Liver Int (2017) 37:936–49. 10.1111/liv.13435 28371239

[80] Parker HM Johnson NA Burdon CA Cohn JS O’Connor HT George J Omega-3 supplementation and non-alcoholic fatty liver disease: a systematic review and meta-analysis. J Hepatol (2012) 56:944–51. 10.1016/j.jhep.2011.08.018 22023985

[81] Yan JH Guan BJ Gao HY Peng XE Omega-3 polyunsaturated fatty acid supplementation and non-alcoholic fatty liver disease: a meta-analysis of randomized controlled trials. Medicine (2018) 97:e12271. 10.1097/MD.0000000000012271 30212963 PMC6155966

[82] Argo CK Patrie JT Lackner C Henry TD de Lange EE Weltman AL Effects of n-3 fish oil on metabolic and histological parameters in NASH: a double-blind, randomized, placebo-controlled trial. J Hepatol (2015) 62:190–7. 10.1016/j.jhep.2014.08.036 PMC4272639 25195547

[83] Shi KQ Fan YC Liu WY Li LF Traditional Chinese medicines benefit to nonalcoholic fatty liver disease: a systematic review and meta-analysis. Mol Biol Rep (2012) 39:9715–22. 10.1007/s11033-012-1836-0 22718512

[84] Wat E Wang Y Chan K Law HW Koon CM Lau KM An in vitro and in vivo study of a 4-herb formula on the management of diet-induced metabolic syndrome. Phytomedicine (2018) 42:112–25. 10.1016/j.phymed.2018.03.028 29655677

[85] Yin F Li L Chen Y Lu T Li W Cai B Quality control of processed Crataegi Fructus and its medicinal parts by ultra high performance liquid chromatography with electrospray ionization tandem mass spectrometry. J Separation Sci (2015) 38:2630–9. 10.1002/jssc.201500021 26009877

[86] Yan H Gao YQ Zhang Y Wang H Liu GS Lei JY Chlorogenic acid alleviates autophagy and insulin resistance by suppressing JNK pathway in a rat model of nonalcoholic fatty liver disease. J Biosci (2018) 43:287–94. 10.1007/s12038-018-9746-5 29872017

[87] Lin YN Chang HY Wang CC Chu FY Shen HY Chen CJ Oleanolic acid inhibits liver X receptor alpha and pregnane X receptor to attenuate ligand-induced lipogenesis. J Agric Food Chem (2018) 66:10964–76. 10.1021/acs.jafc.8b03372 30351048

[88] Gamede M Mabuza L Ngubane P Khathi A Plant-derived oleanolic acid ameliorates markers associated with non-alcoholic fatty liver disease in a diet-induced pre-diabetes rat model. *Diabetes* . Metab Syndrome Obes: Targets Ther (2019) 12:1953–62. 10.2147/DMSO.S218626 PMC6778448 31632109

[89] Ding C Zhao Y Shi X Zhang N Zu G Li Z New insights into salvianolic acid A action: Regulation of the TXNIP/NLRP3 and TXNIP/ChREBP pathways ameliorates HFD-induced NAFLD in rats. Sci Rep (2016) 6:28734. 10.1038/srep28734 27345365 PMC4922017

[90] Wang W Xu AL Li ZC Li Y Xu SF Sang HC Combination of probiotics and Salvia miltiorrhiza polysaccharide alleviates hepatic steatosis via gut microbiota modulation and insulin resistance improvement in high fat-induced NAFLD Mice. Diabetes Metab J (2019) 44:336–48. 10.4093/dmj.2019.0042 PMC7188963 31950772

[91] Huang L Ding W Wang MQ Wang ZG Chen HH Chen W Tanshinone IIA ameliorates non-alcoholic fatty liver disease through targeting peroxisome proliferator-activated receptor gamma and toll-like receptor 4. J Int Med Res (2019) 47:5239–55. 10.1177/0300060519859750 PMC6833399 31378113

[92] Li XX Lu XY Zhang SJ Chiu AP Lo LH Largaespada DA Sodium tanshinone IIA sulfonate ameliorates hepatic steatosis by inhibiting lipogenesis and inflammation. Biomed Pharmacother (2019) 111:68–75. 10.1016/j.biopha.2018.12.019 30576936

[93] Wang YC Jin QM Kong WZ Chen J Protective effect of salvianolic acid B on NASH rat liver through restoring intestinal mucosal barrier function. Int J Clin Exp Pathol (2015) 8:5203–9. PMC4503090 26191218

[94] Shan SS Kai W Ke M Li B Wei LH An insoluble polysaccharide from the sclerotium of Poria cocos improves hyperglycemia, hyperlipidemia and hepatic steatosis in ob/ob mice via modulation of gut microbiota. Chin J Natural Medicines (2019) 17:3–14. 10.1016/S1875-5364(19)30003-2 30704621

[95] Kim JH Sim HA Jung DY Lim EY Kim YT Kim BJ Poria cocus wolf extract ameliorates hepatic steatosis through regulation of lipid metabolism, inhibition of ER stress, and activation of autophagy via AMPK activation. Int J Mol Sci (2019) 20:4801–17. 10.3390/ijms20194801 PMC6801774 31569635

[96] Li S Wang L Du Z Jin S Song C Jia S Identification of the lipid-lowering component of triterpenes from Alismatis rhizoma based on the MRM-based characteristic chemical profiles and support vector machine model. Anal Bioanal Chem (2019) 411:3257–68. 10.1007/s00216-019-01818-x 31089788

[97] Meng Q Duan XP Wang CY Liu ZH Sun PY Huo XK Alisol B 23-acetate protects against non-alcoholic steatohepatitis in mice via farnesoid X receptor activation. Acta Pharmacol Sin (2017) 38:69–79. 10.1038/aps.2016.119 27773935 PMC5220543

[98] Zeng L Tang W Yin J Feng L Li Y Yao X Alisol A 24-acetate prevents hepatic steatosis and metabolic disorders in HepG2 cells. Cell Physiol Biochem (2016) 40:453–64. 10.1159/000452560 27889747

[99] Wu C Jing M Yang L Jin L Ding Y Lu J Alisol A 24-acetate ameliorates nonalcoholic steatohepatitis by inhibiting oxidative stress and stimulating autophagy through the AMPK/mTOR pathway. Chemico-Biol Interact (2018) 291:111–9. 10.1016/j.cbi.2018.06.005 29883724

[100] Law BYK Mo JF Wong VKW Autophagic effects of Chaihu (dried roots of Bupleurum Chinense DC or Bupleurum scorzoneraefolium WILD). Chin Med (2014) 9:1–8. 10.1186/1749-8546-9-21 25228909 PMC4165614

[101] Xia Q Han LW Zhang Y He QX Zhang SS Gao JJ Study on liver protection and hepatotoxicity of saikosaponin a based on zebrafish model. China J Chin Mater Med (2019) 44:2662–6. 10.19540/j.cnki.cjcmm.20190520.205 31359674

[102] Meng SX Liu Q Tang YJ Wang WJ Zheng QS Tian HJ A recipe composed of chinese herbal active components regulates hepatic lipid metabolism of NAFLD in vivo and in vitro. BioMed Res Int (2016) 2016:1026852. 10.1155/2016/1026852 27069915 PMC4812184

[103] Song MY Jung HW Kang SY Park Y-K Atractylenolide III enhances energy metabolism by increasing the SIRT-1 and PGC1α expression with AMPK phosphorylation in C2C12 mouse skeletal muscle cells. Biol Pharm Bull (2017) 40:339–44. 10.1248/bpb.b16-00853 28250276

[104] Mei L Tang Y Li M Yang P Liu Z Yuan J Co-administration of cholesterol-lowering probiotics and anthraquinone from Cassia obtusifolia L. ameliorate non-alcoholic fatty liver. PloS One (2015) 10:e0138078. 10.1371/journal.pone.0138078 26375281 PMC4573521

[105] Jang DS Lee GY Kim YS Lee YM Kim C-S Yoo JL Anthraquinones from the seeds of Cassia tora with inhibitory activity on protein glycation and aldose reductase. Biol Pharm Bull (2007) 30:2207–10. 10.1248/bpb.30.2207 17978503

[106] Jia X Iwanowycz S Wang J Saaoud F Yu F Wang Y Emodin attenuates systemic and liver inflammation in hyperlipidemic mice administrated with lipopolysaccharides. Exp Biol Med (2014) 239:1025–35. 10.1177/1535370214530247 PMC4988953 24740873

[107] Wang S Li X Guo H Yuan Z Wang T Zhang L Emodin alleviates hepatic steatosis by inhibiting sterol regulatory element binding protein 1 activity by way of the calcium/calmodulin-dependent kinase kinase-AMP-activated protein kinase-mechanistic target of rapamycin-p70 ribosomal S6 kinase signaling pathway. Hepatol Res (2017) 47:683–701. 10.1111/hepr.12788 27492505

[108] Yan C Zhang Y Zhang X Aa J Wang G Xie Y Curcumin regulates endogenous and exogenous metabolism via Nrf2-FXR-LXR pathway in NAFLD mice. Biomed Pharmacother (2018) 105:274–81. 10.1016/j.biopha.2018.05.135 29860219

[109] Feng D Zou J Su D Mai H Zhang S Li P Curcumin prevents high-fat diet-induced hepatic steatosis in ApoE–/– mice by improving intestinal barrier function and reducing endotoxin and liver TLR4/NF-κB inflammation. Nutr Metab (2019) 16:1–11. 10.1186/s12986-019-0410-3 PMC6858759 31788011

[110] Shi T Wu L Ma W Ju L Bai M Chen X Nonalcoholic fatty liver disease: pathogenesis and treatment in traditional Chinese medicine and western medicine. Evidence-Based Complement Altern Med (2020) 2020:8749564–79. 10.1155/2020/8749564 PMC6969649 31998400

[111] Zhang SJ Chen ZX Jiang KP Cheng YH Gu YL The effect of QuYuHuaTanTongLuo Decoction on the non-alcoholic steatohepatitis. Complement Therapies Med (2008) 16:192–8. 10.1016/j.ctim.2007.08.004 18638709

[112] Cai Y Liang Q Chen W Chen M Chen R Zhang Y Evaluation of HuoXueHuaYu therapy for nonalcoholic fatty liver disease: a systematic review and meta-analysis of randomized controlled trial. BMC Complement Altern Med (2019) 19:178–88. 10.1186/s12906-019-2596-3 PMC6642602 31324247

[113] Song HY Zhang L Pan JL Yang LL Ji G Bioactivity of five components of Chinese herbal formula Jiangzhi granules against hepatocellular steatosis. J Integr Med (2013) 11:262–8. 10.3736/jintegrmed2013034 23867244

[114] Pan J Wang M Song H Wang L Ji G The efficacy and safety of traditional chinese medicine (jiang zhi granule) for nonalcoholic Fatty liver: a multicenter, randomized, placebo-controlled study. Evidence-Based Complement Altern Med (2013) 2013:965723–30. 10.1155/2013/965723 PMC3867828 24369486

[115] Hopkins AL Network pharmacology: the next paradigm in drug discovery. Nat Chem Biol (2008) 4:682–90. 10.1038/nchembio.118 18936753

[116] Zheng Y Wang M Zheng P Tang X Ji G Systems pharmacology-based exploration reveals mechanisms of anti-steatotic effects of Jiang Zhi Granule on non-alcoholic fatty liver disease. Sci Rep (2018) 8:1–12. 10.1038/s41598-018-31708-8 30209324 PMC6135841

[117] Wang M Sun S Wu T Zhang L Song H Hao W Inhibition of LXR/SREBP-1c-mediated hepatic steatosis by Jiang-Zhi granule. Evidence-Based Complement Altern Med (2013) 2013:584634–43. 10.1155/2013/584634 PMC3670567 23762146

[118] Yang L Wang M Liu T Song H Li D Zheng P Effects of Chinese herbal medicine Jiangzhi Granule on expressions of liver X receptor α and sterol regulatory element-binding protein-1c in a rat model of non-alcoholic fatty liver disease. J Chin Integr Med (2011) 9:998–1004. 10.3736/jcim20110911 21906525

[119] Higuchi N Kato M Shundo Y Tajiri H Tanaka M Yamashita N Liver X receptor in cooperation with SREBP-1c is a major lipid synthesis regulator in nonalcoholic fatty liver disease. Hepatol Res (2008) 38:1122–9. 10.1111/j.1872-034X.2008.00382.x 18684130

[120] Li Y Xu S Mihaylova MM Zheng B Hou X Jiang B AMPK phosphorylates and inhibits SREBP activity to attenuate hepatic steatosis and atherosclerosis in diet-induced insulin-resistant mice. Cell Metab (2011) 13:376–88. 10.1016/j.cmet.2011.03.009 PMC3086578 21459323

[121] Zheng YY Wang M Shu XB Zheng PY Ji G Autophagy activation by Jiang Zhi Granule protects against metabolic stress-induced hepatocyte injury. World J Gastroenterol (2018) 24:992–1003. 10.3748/wjg.v24.i9.992 29531463 PMC5840474

[122] Yang L Zhou Y Song H Zheng P Jiang-Zhi granules decrease sensitivity to low-dose CCl 4 induced liver injury in NAFLD rats through reducing endoplasmic reticulum stress. BMC Complement Altern Med (2019) 19:1–11. 10.1186/s12906-019-2641-2 31438932 PMC6704726

[123] Zhao Y Pan Y Yang Y Batey R Wang J Li Y Treatment of rats with Jiangzhi Capsule improves liquid fructose-induced fatty liver: modulation of hepatic expression of SREBP-1c and DGAT-2. J Trans Med (2015) 13:174–85. 10.1186/s12967-015-0529-6 PMC4467629 26031670

[124] Zhou L Yang J Wang M Gao Y Zhang S Sun Y Effect of Dachaihu decoction on non-alcoholic fatty liver disease model rats induced by a high-fat high-sugar diet. J Trad Chin Med Sci (2018) 5:390–9. 10.1016/j.jtcms.2018.10.001

[125] Zhang M Yuan Y Wang Q Li X Men J Lin M The Chinese medicine Chai Hu Li Zhong Tang protects against non-alcoholic fatty liver disease by activating AMPKα. Biosci Rep (2018) 38:BSR20180644. 10.1042/BSR20180644 30291215 PMC6239269

[126] Cheng F Ma C Wang X Zhai C Wang G Xu X Effect of traditional Chinese medicine formula Sinisan on chronic restraint stress-induced nonalcoholic fatty liver disease: a rat study. BMC Complement Altern Med (2017) 17:203–12. 10.1186/s12906-017-1707-2 PMC5383977 28388904

[127] Zhu F Li YM Feng TT Wu Y Zhang HX Jin GY Freeze-dried Si-Ni-San powder can ameliorate high fat diet-induced non-alcoholic fatty liver disease. World J Gastroenterol (2019) 25:3056–68. 10.3748/wjg.v25.i24.3056 PMC6603807 31293341

[128] Paredes-Turrubiarte G González-Chávez A Pérez-Tamayo R Salazar-Vázquez BY Hernández VS Garibay-Nieto N Severity of non-alcoholic fatty liver disease is associated with high systemic levels of tumor necrosis factor alpha and low serum interleukin 10 in morbidly obese patients. Clin Exp Med (2016) 16:193–202. 10.1007/s10238-015-0347-4 25894568

[129] Kakino S Ohki T Nakayama H Yuan X Otabe S Hashinaga T Pivotal role of TNF-α in the development and progression of nonalcoholic fatty liver disease in a murine model. Hormone Metab Res (2018) 50:80–7. 10.1055/s-0043-118666 28922680

[130] Mitchell WJ Physiology of carbohydrate to solvent conversion by clostridia. Adv Microbial Physiol (1998) 39:31–130. 10.1016/S0065-2911(08)60015-6 9328646

[131] Ter Horst KW Serlie MJ Fructose consumption, lipogenesis, and non-alcoholic fatty liver disease. Nutrients (2017) 9:981. 10.3390/nu9090981 PMC5622741 28878197

[132] Jensen T Abdelmalek MF Sullivan S Nadeau KJ Green M Roncal C Fructose and sugar: A major mediator of non-alcoholic fatty liver disease. J Hepatol (2018) 68:1063–75. 10.1016/j.jhep.2018.01.019 PMC5893377 29408694

[133] Zhang Y Tang K Deng Y Chen R Liang S Xie H Effects of shenling baizhu powder herbal formula on intestinal microbiota in high-fat diet-induced NAFLD rats. Biomed Pharmacother (2018) 102:1025–36. 10.1016/j.biopha.2018.03.158 29710519

[134] Nobili V Putignani L Mosca A Del Chierico F Vernocchi P Alisi A Bifidobacteria and lactobacilli in the gut microbiome of children with non-alcoholic fatty liver disease: which strains act as health players? Arch Med Sci: AMS (2018) 14:81–7. 10.5114/aoms.2016.62150 PMC5778421 29379536

[135] Yin J Luo Y Deng H Qin S Tang W Zeng L Hugan Qingzhi medication ameliorates hepatic steatosis by activating AMPK and PPARα pathways in L02 cells and HepG2 cells. J Ethnopharmacol (2014) 154:229–39. 10.1016/j.jep.2014.04.011 24735863

[136] Tang W Zeng L Yin J Yao Y Feng L Yao X Hugan Qingzhi exerts anti-inflammatory effects in a rat model of nonalcoholic fatty liver disease. Evidence-Based Complement Altern Med (2015) 2015:810369–81. 10.1155/2015/810369 PMC4471380 26146507

[137] Tang W Yao X Xia F Yang M Chen Z Zhou B Modulation of the gut microbiota in rats by hugan qingzhi tablets during the treatment of high-fat-diet-induced nonalcoholic fatty liver disease. Oxid Med Cell Longevity (2018) 2018:7261619–32. 10.1155/2018/7261619 PMC6323444 30671174

[138] Liang S Zhang Y Deng Y He Y Liang Y Liang Z The potential effect of Chinese herbal formula hongqijiangzhi fang in improving NAFLD: focusing on NLRP3 inflammasome and gut microbiota. Evidence-Based Complement Altern Med (2018) 2018:5378961–73. 10.1155/2018/5378961 PMC5841032 29675053

[139] Zhu Y Huang JJ Zhang XX Yan Y Yin XW Ping G Qing gan zi shen tang alleviates adipose tissue dysfunction with up-regulation of sirt1 in spontaneously hypertensive rat. Biomed Pharmacother (2018) 105:246–55. 10.1016/j.biopha.2018.05.022 29859467

[140] Lee H Shim EH Lee MS Myung CS Traditional medicine, Sobokchukeo−Tang, modulates the inflammatory response in adipocytes and macrophages. Mol Med Rep (2017) 15:117–24. 10.3892/mmr.2016.6005 PMC5355692 27959437

[141] Mendez-Sanchez N Cruz-Ramon VC Ramirez-Perez OL Hwang JP Barranco-Fragoso B Cordova-Gallardo J New aspects of lipotoxicity in nonalcoholic steatohepatitis. Int J Mol Sci (2018) 19:2034–54. 10.3390/ijms19072034 PMC6073816 30011790

[142] Cui Y Chang R Zhang T Zhou X Wang Q Gao H Chinese Herbal Formula (CHF03) attenuates non-alcoholic fatty liver disease (NAFLD) through inhibiting lipogenesis and anti-oxidation mechanisms. Front Pharmacol (2019) 10:1190–201. 10.3389/fphar.2019.01190 31680967 PMC6803500

[143] Perry B Zhang J Saleh T Wang Y Liuwei Dihuang, a traditional Chinese herbal formula, suppresses chronic inflammation and oxidative stress in obese rats. J Integr Med (2014) 12:447–54. 10.1016/S2095-4964(14)60044-3 25292344

[144] Akbal E Koçak E Taş A Yüksel E Köklü S Visfatin levels in nonalcoholic fatty liver disease. J Clin Lab Anal (2012) 26:115–9. 10.1002/jcla.21491 PMC6807497 22467327

[145] Elkabany ZA Hamza RT Ismail EAR Elsharkawy A Yosry A Musa S Serum visfatin level as a noninvasive marker for nonalcoholic fatty liver disease in children and adolescents with obesity: relation to transient elastography with controlled attenuation parameter. Eur J Gastroenterol Hepatol (2020) 32:1008–16. 10.1097/MEG.0000000000001608 31834057

[146] Li S Fan TP Jia W Lu A Zhang W Network pharmacology in traditional chinese medicine. Evidence-Based Complement Altern Med (2014) 2014:138460–1. 10.1155/2014/138460 PMC3953584 24707305

[147] Li J Lu C Jiang M Niu X Guo H Li L Traditional chinese medicine-based network pharmacology could lead to new multicompound drug discovery. Evidence-Based Complement Altern Med (2012) 2012:149762–72. 10.1155/2012/149762 PMC3541710 23346189

[148] Luo J Ren Y Gu H Wu Y Wang Y dTGS: method for effective components identification from traditional Chinese medicine formula and mechanism analysis. Evidence-Based Complement Altern Med (2013) 2013:840427–35. 10.1155/2013/840427 PMC3878852 24454516

[149] Cui JX Cui HF Yang MR Du SY Li JF Li YX Tongue coating microbiome as a potential biomarker for gastritis including precancerous cascade. Protein Cell (2019) 10:496–509. 10.1007/s13238-018-0596-6 30478535 PMC6588651

[150] Huo X Lu F Qiao L Li G Zhang Y A component formula of Chinese medicine for hypercholesterolemia based on virtual screening and biology network. Evidence-Based Complement Altern Med (2018) 2018:1854972–82. 10.1155/2018/1854972 PMC6046189 30050582

